# Reference values of four measures of craniocervical stability using upright dynamic magnetic resonance imaging

**DOI:** 10.1007/s11547-023-01588-8

**Published:** 2023-01-30

**Authors:** Leslie L. Nicholson, Prashanth J. Rao, Matthew Lee, Tsz Ming Wong, Regen Hoi Yan Cheng, Cliffton Chan

**Affiliations:** 1 School of Medical Sciences, The University of Sydney, Camperdown, NSW Australia; 2 Macquarie University Hospital, Macquarie Park, NSW Australia; 3 Faculty of Medicine and Health, Macquarie University, Macquarie Park, NSW Australia; 4 Radiology, Western Imaging Group, Blacktown, NSW Australia; 5 Department of Rehabilitation Sciences, Hong Kong Polytechnic University, Hung Hom, Kowloon, Hong Kong

**Keywords:** Craniocervical instability, Hypermobility, Reliability, Cutoffs, Atlanto-axial joint, Atlanto-occipital joint

## Abstract

**Purpose:**

To establish reference ranges for four most commonly used diagnostic measures of craniocervical instability (CCI) in three cervical sagittal positions. This necessitated development of a reliable measurement protocol using upright, dynamic MRI (udMRI), to determine differences in the extent of motion between positions, and whether age and sex correlate with these measures.

**Materials and Methods:**

Deidentified udMRIs of 50 adults, referred for reasons other than CCI, were captured at three positions (maximal flexion, maximal extension and neutral). Images were analyzed, providing measures of basion-axial interval, basion-axial angle, basion-dens interval (BDI) and the Grabb–Oakes line (GOL) for all three positions (12 measures per participant). All measures were independently recorded by a radiologist and neurosurgeon to determine their reliability. Descriptive statistics, correlations, paired and independent t-tests were used. Mean (± 2 SD) identified the reference range for all four measures at each craniocervical position.

**Results:**

The revised measurement protocol produced inter-rater reliability indices of 0.69–0.97 (moderate–excellent). Fifty adults’ (50% male; mean age 41.2 years (± 9.7)) reference ranges for all twelve measures were reported. Except for the BDI and GOL when moving between neutral and full flexion, significant extents of movement were identified between the three craniocervical positions for all four measures (*p* ≤ 0.005). Only a minor effect of age was found.

**Conclusions:**

This is the first study to provide a rigorous standardized protocol for four diagnostic measures of CCI. Reference ranges are established at mid and ends of sagittal cervical range corresponding to where exacerbations of signs and symptoms are commonly reported.

## Introduction

Craniocervical stability relies on the integrity of the craniocervical junction which comprises the occiput, atlas and axis that form the occipitoatlantoaxial joint complex [1]. Despite enclosing the brainstem, spinal cord, cranial nerves, C1 and C2 spinal nerve roots and vertebral arteries [1], this junction permits extensive motion particularly in the sagittal and horizontal planes [2]. Its complex kinematics and dynamic stability are conferred by the integrity of specialized ligaments and membranes in addition to joint congruity [3]. Ligamentous laxity and/or bony deficits can result in craniocervical instability (CCI) and allow excessive excursion that jeopardizes the functioning of the central nervous system and cranial nerves [2]. Despite its musculoskeletal origin, CCI can result in debilitating neurological complications.

Consequences of CCI can seriously impact quality of life. Basilar invagination and/or vertebral artery occlusion can result in ventral compression of neural tissues and obstruction of cerebrospinal flow and arterial supply [4, 5]. Reported symptoms although controversial range from headache, vertigo, perceived instability and sensorimotor dysfunction to impaired vision, dyspnea and dysautonomia [5–8]. However, patients present heterogeneously and may be referred for diagnostic procedures not specific to CCI.

The pathophysiology of CCI is variable. A range of conditions are associated with CCI including, but not limited to, head/neck injury [9], arthritic conditions [10, 11], genetic disorders [12] and heritable disorders of connective tissue [13–15]. Despite known associations, the condition-specific prevalence of CCI is inconsistently reported. For example, 10–70% of patients with rheumatoid arthritis are diagnosed with CCI [10, 16], 8–63% of those with Down syndrome [12, 17] and 25–37% with osteogenesis imperfecta [18, 19]. The methodological variability between studies, particularly in the classification of severity and diagnostic criteria of CCI utilized, highlights the difficulties in determining its prevalence and potential impact at both the individual and population levels.

Clinical diagnosis of CCI is confirmed by positive radiological findings. These are well defined for traumatic conditions but less so for nontraumatic etiology. Patients with CCI report symptoms and demonstrate signs of ventral compression during head and neck movements [6, 7, 9, 20–22]. Although the common static imaging techniques, such as erect radiography and recumbent MRI, might suffice to detect overt subluxations or neuroanatomical abnormalities [23, 24], signs of mild instability or positional symptoms may be difficult to discern from images taken in a neutral or unloaded head-on-neck position. The presence of provocative positions in CCI, notably flexion, suggests that subluxations and neuroanatomical distortions may be positional and therefore be best diagnosed with functional radiological investigations.

Upright dynamic MRI (udMRI) may substantially enhance diagnosis of CCI [25]. Preliminary evidence suggests that udMRI demonstrates superior diagnostic efficacy compared to static supine or upright imaging. Indeed, plain film and computed tomography imaging provide less accurate and valid measures when assessing the extent of soft tissue pathologies in this area [9, 20, 22]. However, the interpretation of craniocervical measures on udMRI remains unstandardized. Normative ranges in neutral are variously reported [24, 26–28], resulting in different cutoff criteria to define the presence of CCI, while normative ranges in maximum flexion and extension are lacking. These gaps render the establishment and validation of the udMRI-specific cutoff values impossible. Reference ranges of CCI measures on udMRI must be determined to enable evidence-based, and objective diagnosis and classification of CCI and to facilitate research addressing management.

The objectives of this exploratory study were fivefold. Using four routine radiological measures, we aimed to determine (i) a reliable measurement protocol to detect the presence of CCI using udMRI, (ii) the reference ranges in three positions (maximal flexion, maximal extension and neutral), (iii) whether differences exist in the extent of motion between neutral to flexion and neutral to extension, (iv) the correlation of age and sex with the measures and v) the proportion of false-positive identifications of CCI.

## Methods

### Participant recruitment and imaging equipment

Staff of a medical diagnostic imaging center (Western Imaging Group, NSW, Australia) provided deidentified, udMRI images of the craniocervical region of male and female patients 18 years or over. The MRI used in this study was the FONAR UPRIGHT® Multi-Position™ MRI (0.6 Tesla). All were referred for reasons other than CCI. Images of patients who were referred with head or neck trauma, whiplash associated disorder, rheumatological conditions of the craniocervical spine or a hereditary disorder of connective tissue (including but not limited to Ehlers-Danlos Syndrome, Marfan or Loeys-Dietz Syndromes or Osteogenesis Imperfecta) were excluded from the study.

T2 images were captured in the midsagittal plane representing the neutral cervical position (repetition time (TR): 1602 (or 2976 depending on the number of slices used in the original scan) and time to echo (TE): 120), the maximal flexion position (TR: 2082 and TE: 160) and the maximal extension position (TR: 2082 and TE: 160).

### Procedure

**Fig. 1:**
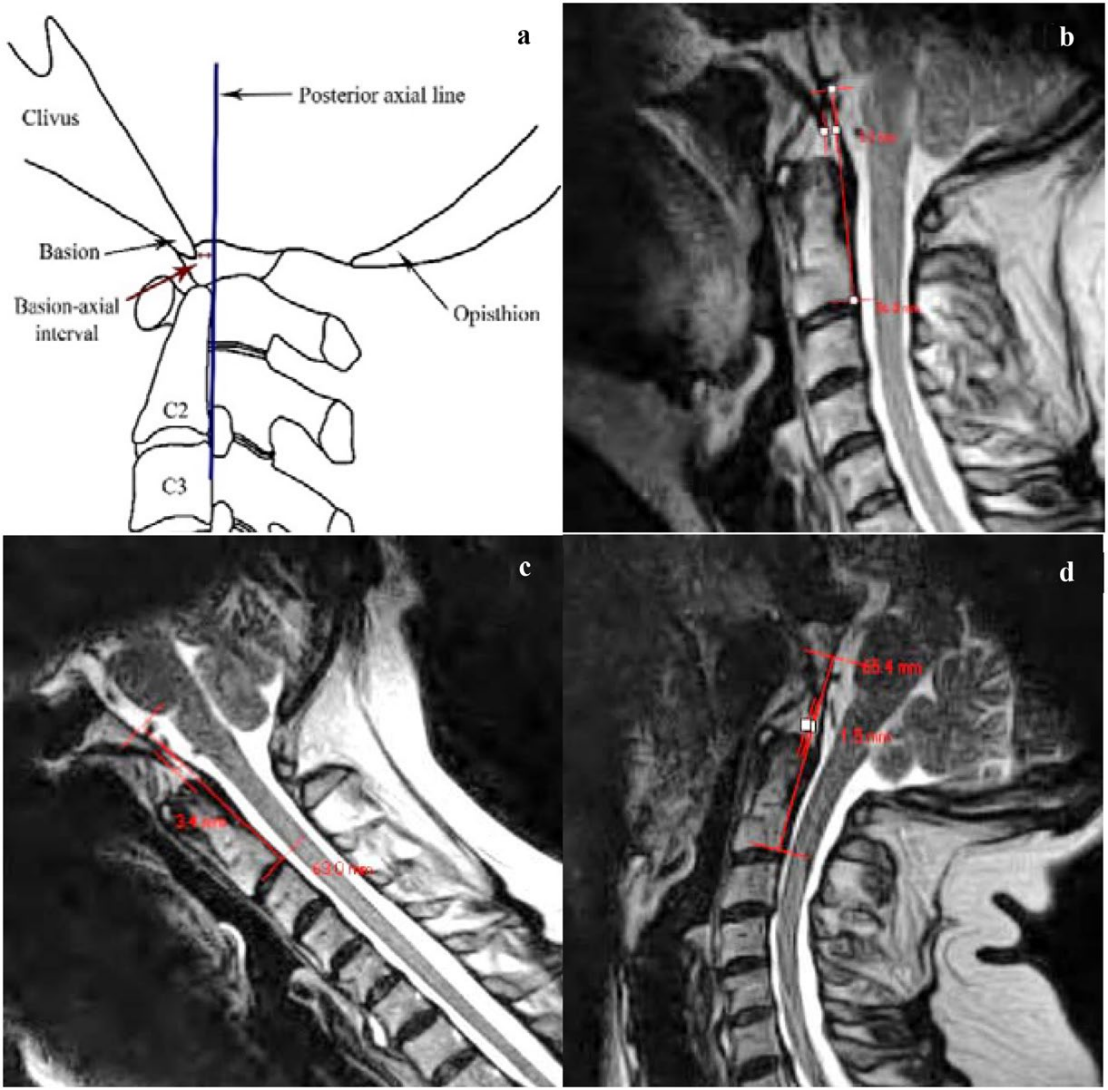
**a** Schematic representation of the basion-axial interval (BAI), also known as the horizontal Harris measurement. Increase in BAI suggests anterior translation of the cranium relative to the axis [29, 30]. The posterior axial line is drawn through the posteroinferior and the posterior most margins of the vertebral body of C2, regardless of the orientation of the posterosuperior aspect of the odontoid. Reported normal values ≤ 12 mm [29]. **b** At a neutral cervical spine position, the proposed BAI reference range is 0.5–8.9 mm. **c** At the maximum cervical flexion position, the proposed reference range is 0.7–10.3 mm. **d** Maximum cervical extension position, the proposed reference range is − 0.6–7.0 mm. (all images lossy compressed 11%)

**Fig. 2:**
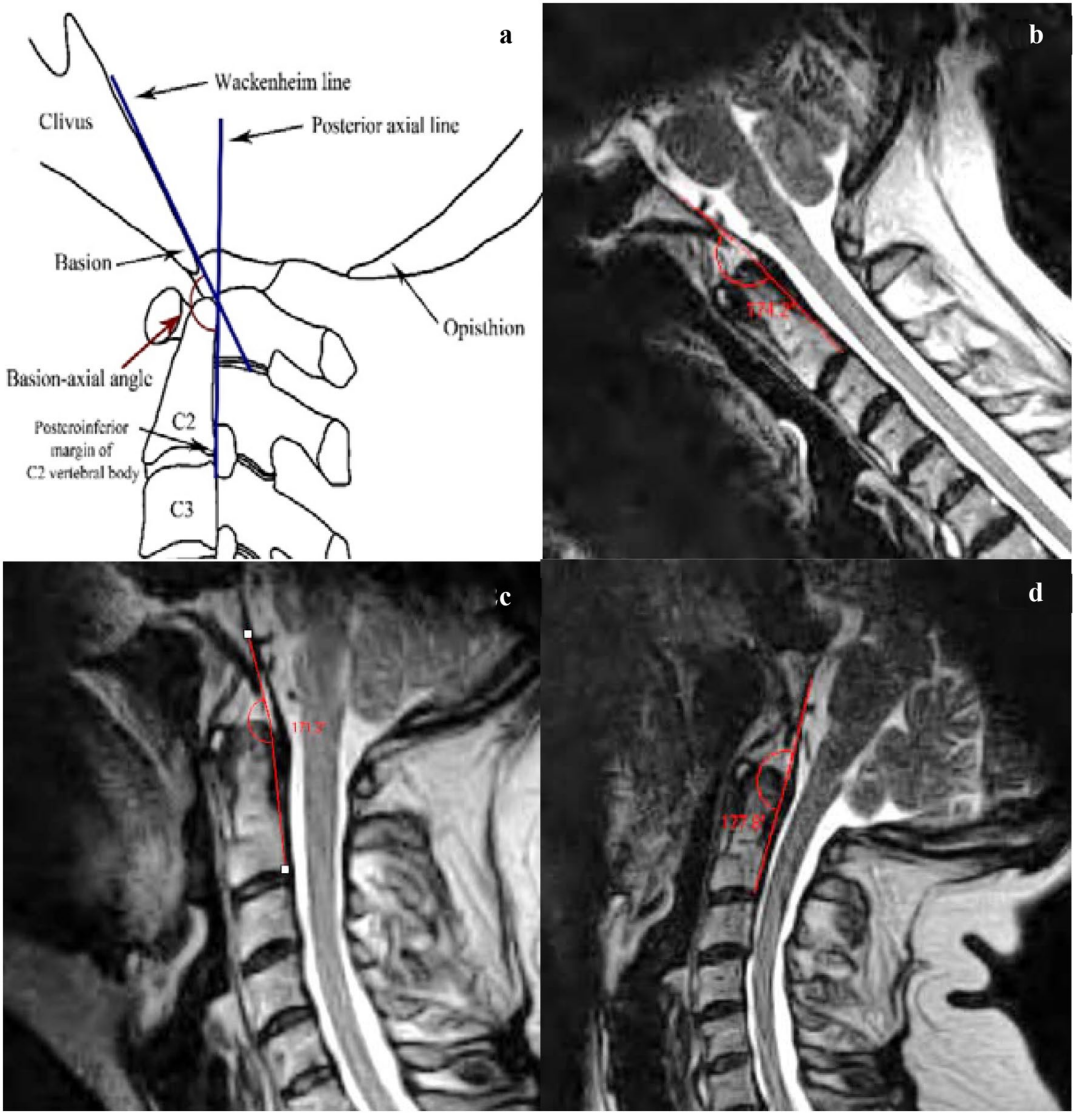
**a** Schematic representation of the basion-axial angle (BAA), also known as the clivo-axial angle (CXA) or clivo-canal angle has suggested normal values > 135° [29]. The Wackenheim line is drawn along the dorsal surface of the lower clivus and extrapolated posteroinferiorly across the superior aspect of the dens tangentially [37]. If the basion curves inferiorly, the line is extended from the middle of the clivus. The posterior axial line is drawn through the posteroinferior and the posterior most margins of the vertebral body of C2, regardless of the odontoid process. The ventral angle (in degrees) of intersection of the two lines is the basion-axial angle. Reduction in BAA suggests increased kyphosis and deformative strain of the brainstem and upper spinal cord [29]. **b** At a neutral cervical spine position, the proposed BAA reference range is 128–169°. **c** At the maximum cervical flexion position, the proposed BAA reference range is 126–165°. **d** Maximum cervical extension position, the proposed BAA reference range is 139–184°. (all images lossy compressed 11%)

**Fig. 3:**
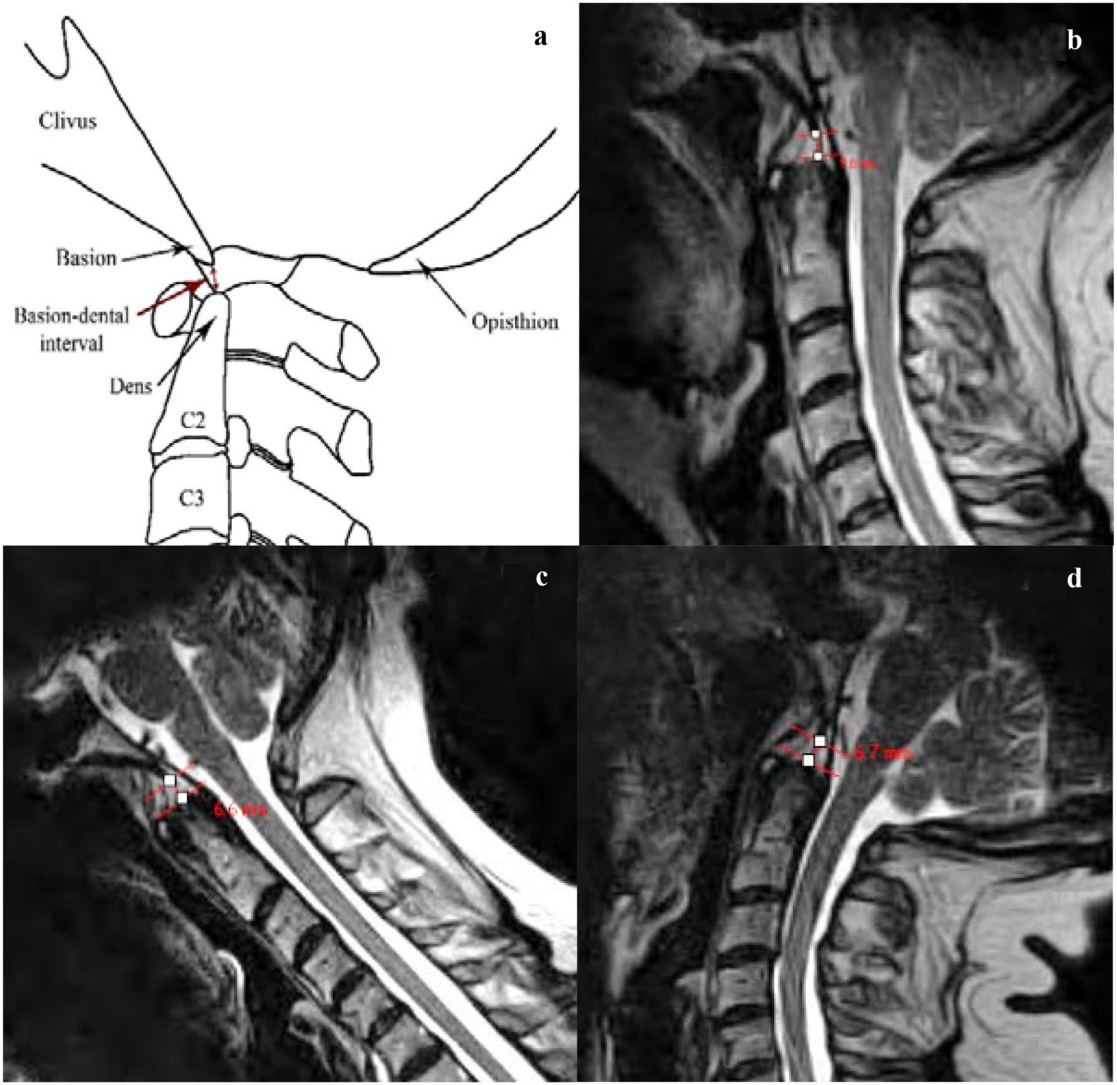
**a** Schematic representation of the basion-dental interval (BDI), which is the minimum distance (in millimeters) from the posteroinferior tip of the basion to the superior aspect of the dens. It has suggested normal values ≤ 12 mm [30]. Increase in BDI or the vertical Harris measurement suggests potential occipito-atlantal instability [26, 38]. **b** At a neutral cervical spine position, the proposed BDI reference range is 2.0–8.0 mm. **c** At the maximum cervical flexion position, the proposed BDI reference range is 1.8–8.2 mm. **d** Maximum cervical extension position, the proposed BDI reference range is 2.4–8.8 mm. (all images lossy compressed 11%)

**Fig. 4:**
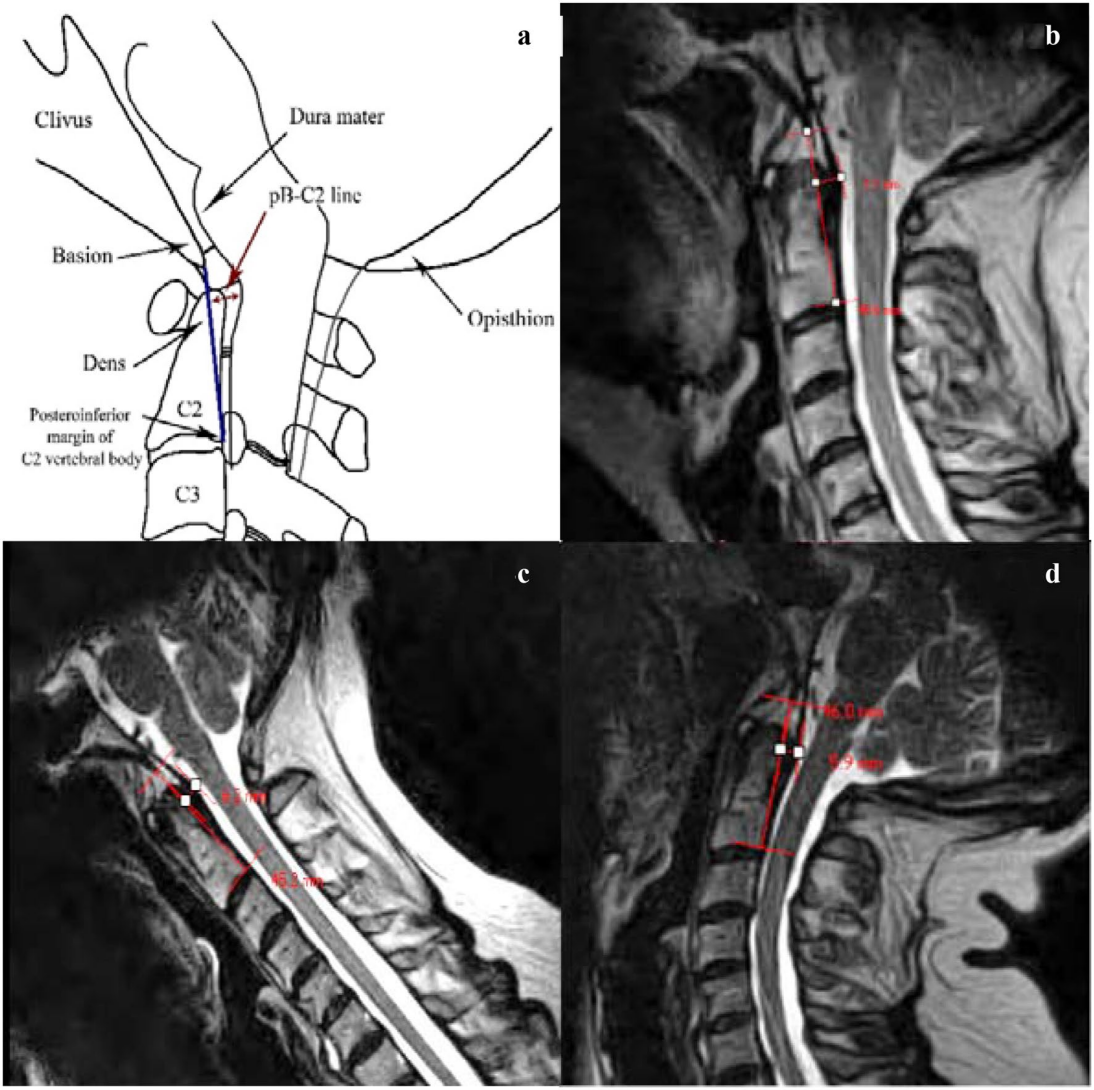
**a** Schematic representation of the Grabb–Oakes line (GOL), also known as the Grabb–Mapstone–Oaks measurement is described as a line drawn from the tip of the basion to the infero-posterior aspect of the body of C2. pB-C2 is the perpendicular distance between this line and the ventral cervicomedullary dura [Wholey]. It has suggested normal values of < 9 mm [29]. Increase in length suggests increased risk of ventral compression of the brainstem and upper spinal cord [29, 39]. **b** At a neutral cervical spine position, the proposed GOL reference range is 4.2–10.2 mm. **c** At the maximum cervical flexion position, the proposed GOL reference range is 3.8–10.6 mm. **d** Maximum cervical extension position, the proposed GOL reference range is 2.7–9.1 mm. (all images lossy compressed 11%)

Three images were extracted for measurement from each patient’s file. These were the midsagittal images captured at maximum cervical flexion, extension and at neutral (mid-way between). The measures chosen to determine craniocervical motion were the (i) basion-axial interval (BAI) also known as the horizontal Harris measurement, (ii) basion-axial angle (BAA) also called the clivo-axial angle, (iii) basion-dens interval (BDI) also known as the vertical Harris measurement and (iv) Grabb–Oakes line (GOL) also known as the Grabb–Mapstone–Oaks measurement. The deidentified scans were reviewed on a medical image viewer (Voyager PACS Intellirad Solutions Pty Ltd, Australia). The program permitted the examiner to mark up the images, automatically calculating the intervals and angles required. The standardized protocol for each measurement including normal reported values is detailed in Figs. 1, 2, 3 and 4.

We aimed to utilize 50 participants’ udMRI for this exploratory study, similar sample size to other investigative MRI studies related to craniocervical measures [7, 20]. To determine the inter-tester reliability of the four measures used in the protocol, two of the investigators (senior radiologist ML and senior neurosurgeon PJR) independently measured these on all three images (neutral and maximal flexion and extension). The diagnostic imaging center provided demographic data only to the chief investigator (LLN) to ensure that those performing the measures were blinded to each patient’s age, sex and reason for referral. A priori, we chose to determine the inter-tester reliability (ICCs) of all four measures in each of the three craniocervical positions for the first 20 participants. If any of the twelve ICCs were not acceptable (< 0.7), the assessors would meet to refine the protocol and remeasure the scans and ICCs would be recalculated. If the inter-rater reliability was deemed acceptable, the measures of the radiologist would be used for the remainder of the analysis as this would be consistent with clinical practice.

Visual inspection of the participants’ measures on the BAI, BAA, BDI and GOL in each of the three sagittal cervical positions was undertaken. To determine the proportion of false-positive identification of radiological evidence of CCI in our cohort, traditional cutoff criteria for normality were used based on previous studies [29, 30].

### Statistical analysis

All data were analyzed using SPSS (v26 IBM NY USA). Descriptive statistics were used to detail demographic data of age, sex and reason for imaging referral. Intraclass correlation coefficients (ICCs) and their 95% confidence intervals (95% CIs) were used to determine the inter-rater reliability of the four radiological measures in the three cervical spine positions. ICC values of < 0.50 indicate poor reliability, 0.50–0.74 indicate moderate reliability, 0.75–0.90 indicate good reliability, and values greater than 0.90 indicate excellent reliability [31].

Whole cohort means, standard deviations and ranges were determined for each of the radiological measures in all three positions to determine reference values. These cutoff values to differentiate normal from abnormal measures were set at two standard deviations above and below the mean as previously recommended [32, 33]. Paired t-tests and Pearson’s correlation coefficients were used to determine whether relative motion differed between the three craniocervical positions for each of the four radiological measures. That is, was there a significant difference between the extent of motion measured between the neutral and maximal flexion positions, and the neutral and maximal extension positions? We set statistical significance level at *p* ≤ 0.005 using Bonferroni correction to account for the multiple hypotheses and to minimize type 1 errors. To determine whether age or sex affects these measures, we used independent samples *t*-tests. Pearson’s correlation (*r*) was used to determine whether any of the measures were associated with age or sex.

Ethical approval was granted from The University of Sydney Human Research Ethics Committee (Approval #2020/284).

## Results

The maximal craniocervical flexion and extension images together with the neutral image in the median sagittal plane of 50 participants were extracted from the series of images captured using udMRI. The mean age (SD, range) of these participants was 41.2 years (± 9.7, 24–69) with no significant age difference between sexes (41.2 years (± 9.3, 24–58) for females; 41.3 years (± 10.3, 27–69) for males (*p* = 0.98)). Fifty percent of the cohort were male. All data were normally distributed. The reasons for referral for udMRI were as follows: 72% cervical radiculopathy, 24% neck pain and 4% cervical myelopathy.

While the ICCs for the first 20 patients revealed that the BAA and GOL measures demonstrated good to excellent reliability, the BAI in neutral and all BDI measures demonstrated unacceptable reliability (< 0.7). The assessors met to discuss individual differences in interpreting the protocol, resolved these and revised the protocol. All ICCs for the first 20 participants were recalculated. Acceptable inter-tester reliability was now demonstrated for all four outcome measures. When performed on all 50 patients, the ICCs ranged between 0.69 (95%CI 0.45–0.82) and 0.97 (95%CI 0.95–0.98).

The mean, standard deviation, range and the limits of “reference values” for each of the radiological measures in all three craniocervical positions (flexion, extension and neutral) are provided in Table 1. The reference range (also provided in Figs. 1, 2, 3 and 4) is the mean ± 2 standard deviations.

**Table 1:** Descriptive statistics of the four radiological measures, with calculated limits indicating reference range (interval between mean − 2 SD to mean + 2 SD)

	Sample statistics		Reference values		
	Mean	SD	Range	Mean − 2 SD	Mean + 2 SD
*Basion-axial interval (BAI)*					
Neutral (mm)	4.7	2.1	1.0−9.5	0.5	8.9
Flexion (mm)	5.5	2.4	0.5–11.0	0.7	10.3
Extension (mm)	3.2	1.9	0.0–8.5	-0.6	7.0
*Basion-axial angle (BAA)*					
Neutral (°)	148.6	10.3	128.0–168.0	128.0	169.2
Flexion (°)	145.8	9.8	125.0–171.0	126.2	165.4
Extension (°)	161.4	11.1	128.0–180.0	139.2	183.6
*Basion-dens interval (BDI)*					
Neutral (mm)^a^	5.0	1.5	3.0–9.0	2.0	8.0
Flexion (mm)^a^	5.0	1.6	2.5–11.0	1.8	8.2
Extension (mm)	5.6	1.6	3.0–9.5	2.4	8.8
*Grabb–Oakes line (GOL)*					
Neutral (mm)^a^	7.2	1.5	4.5–11.0	4.2	10.2
Flexion (mm)^a^	7.2	1.7	2. 5–10.5	3.8	10.6
Extension (mm)	5.9	1.6	3.0–10.5	2.7	9.1

^a^no significant difference between the two BDI measurements (neutral and max. flexion); no significant difference between the two GOL measurements (neutral and max. flexion)

The means of most of the four radiological measures, assessed at neutral and maximal flexion and extension, were significantly different from one another (Table 2). The only measures for which there was no statistical difference (motion between neutral and either flexion or extension) were for the BDI and GOL with negligible mean change when moving from between neutral and flexion.

**Table 2:** Correlations between the measures at each of the craniocervical positions

Measure	*r*	t	Diff	95% CI	*p*
*Basion-axial interval (BAI)*					
Neutral–flexion (mm)	0.77	-3.5	-0.75	-1.18– − 0.31	0.001*
Neutral–extension (mm)	0.69	6.5	1.46	1.01–1.91	0.000*
Flexion–extension (mm)	0.56	7.6	2.21	1.62–2.80	0.000*
*Basion-axial angle (BAA)*					
Neutral–flexion (°)	0.78	2.9	2.78	0.87–4.69	0.005*
Neutral–extension (°)	0.51	-8.6	-12.8	-15.83– − 9.81	0.000*
Flexion–extension (°)	0.48	-10.3	-15.6	-18.63– − 12.57	0.000*
*Basion-dens interval (BDI)*					
Neutral–flexion (mm)	0.71	-0.07	-0.01	-0.31–0.29	0.946
Neutral–extension (mm)	0.61	-3.4	-0.65	-1.03– − 0.27	0.001*
Flexion–extension (mm)	0.61	-3.2	-0.64	-1.04– − 0.24	0.002*
*Grabb–Oakes line (GOL)*					
Neutral–flexion (mm)	0.69	0.5	0.08	-0.29–0.45	0.656
Neutral–extension (mm)	0.70	7.8	1.35	1.00–1.70	0.000*
Flexion–Extension (mm)	0.45	5.1	1.27	0.77–1.77	0.000*

*r*—Pearson correlation; Diff—mean difference in the measure between the two craniocervical positions

*significant correlation at *p* ≤ 0.005

None of the measures (BAI, BAA, BDI and GOL) were statistically different between male and female participants (all *p*-values ranged from 0.05 to 0.94) in any of the three cervical positions. Hence, no correlation analyses were performed between the four diagnostic measures and sex. Increasing age was associated with decrease in the extent of some of the measures in flexion and neutral but not in extension. Only the BAI in the neutral position was significantly decreased with age (Table 3).

**Table 3:** Correlation of each of the four radiological measures in all three craniocervical positions, with age

	Correlation with age	*p*	Effect size
*Basion-axial interval (BAI)*			
Neutral	− 0.433	0.002*	Medium
Flexion	− 0.375	0.007	Medium
Extension	− 0.270	0.058	Small
*Basion-axial angle (BAA)*			
Neutral	0.385	0.006	Medium
Flexion	0.377	0.007	Medium
Extension	− 0.001	0.992	Negligible
*Basion-dens interval (BDI)*			
Neutral	− 0.184	0.200	Small
Flexion	0.087	0.549	Negligible
Extension	0.013	0.928	Negligible
*Grabb–Oakes line (GOL)*			
Neutral	− 0.383	0.006	Medium
Flexion	− 0.168	0.243	Small
Extension	− 0.209	0.146	Small

*significant correlation at *p* ≤ 0.005

The number of participants without suspected CCI signs or symptoms (as indicated by the referral) in this study who exhibited results that would be considered diagnostic of this condition as determined by traditional cutoff criteria, that is false positives, are presented in Table 4. Of the 50 participants, two exhibited both abnormal BAA and GOL. These were females, aged 25 and 33 years, and both were referred to imaging for “cervical radiculopathy.”

**Table 4:** Number of participants (and proportion of the cohort) whose measurements met the criterion for “abnormal” for each of the four measures in each position

	Criterion for “abnormal” designation	Neutral *n* (%)	Flexion *n* (%)	Extension *n* (%)
BAI	> 12 mm [29]	0	0	0
BAA	≤ 135° [29]	4 (8%)	4 (8%)	1 (2%)
BDI	> 12 mm [30]	0	0	0
GOL	≥ 9 mm [29]	6 (12%)	7 (14%)	3 (6%)

*BAI* basion-axial interval, *BAA* basion-axial angle, *BDI* basion-dens interval, *GOL* Grabb–Oakes line

## Discussion

To our knowledge, this study is one of the largest to analyze common measures associated with diagnosis of craniocervical instability (CCI), using upright dynamic MRI. Given that CCI symptoms may only occur or be exacerbated in end-range positions during daily activities [34], it is pertinent to image the craniocervical region in these ranges. We refined and produced a clinically useful protocol for four commonly used measures in mid and end-range flexion and extension, providing reference ranges outside of which abnormalities may be identified.

**Fig. 5:**
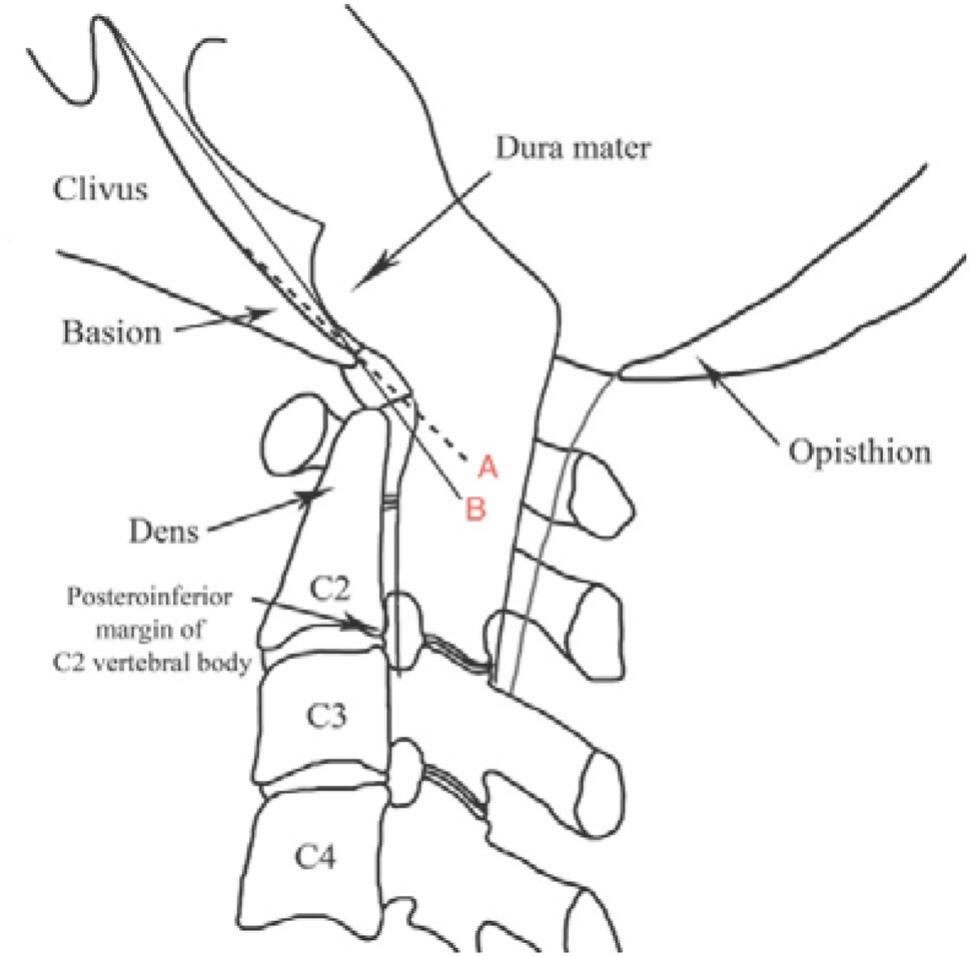
Two interpretations of the Wackenheim line reported in the literature. **A** [35] and **B** [30]. Interpolations of lines: **A** depicts the line generated using only the distal portion of the posterior clivus. **B** depicts the line generated using the inferior and superior aspects of the posterior clivus

Prior to collecting the data, we reviewed the literature to find varying descriptions of the measurements. One of the challenges faced by those taking the measures and then by those interpreting them is the variability in protocols used. Much of the unreliability inherent in the measures reported relates to the lack of detail provided by investigators and the anatomical variability of the bony structures. An example of this is the placement of the Wackenheim line. Harris and colleagues report that some measurements use the rostral slope of the posterior clivus [30]. Other investigators interpolate a line that connects the superior and inferior extents of the posterior aspects of the clivus [35]. These versions will generate different BAA measurements (Fig. 5).

This was clearly evidenced by the initial less-than-acceptable inter-rater reliability (ICC < 0.7) initially calculated for two of the four measures. Consultation was undertaken by the two highly experienced examiners to come to a consensus on the protocol for each measure.

The reference ranges as a proxy for “normal” range for the four measures indicate that significant extents of movement occur between the bony landmarks during upper cervical flexion and extension (Table 2). There was a mean 12% increase in the BAI when moving into flexion, a 9% decrease in the BAA moving into extension and a 15% increase in GOL length when moving into extension. These findings further validate the need to use udMRI to fully investigate and understand the mechanics of symptom production in patients with CCI or symptoms associated with end-range craniocervical positioning.

While sex appeared to have no association with relative motion between the skull, atlas and axis, moderate correlations were found between age and motion at the neutral position for BAI, BAA and GOL and in the flexion position for BAI and BAA. BAI and GOL demonstrated that as people age, the intervals decrease, whereas the BAA increased with age in both the flexion and neutral positions.

Our established reference ranges align with the previously recommended cutoff values for the basion-axial interval [29, 30] (horizontal Harris measurement) and the basion-dental interval [30] (vertical Harris measurement). However, our reference ranges for the basion-axial angle [29, 37] (clivo-axial angle) suggest that the > 135° cutoff to indicate normality may result in over-diagnosis of kyphosis resulting in deformation of the brainstem and upper spinal cord. We recorded measures of ≤ 135° in 2–8% (depending on craniocervical position) of our 50 participants who were not referred with symptoms suggestive of CCI. Similarly, our reference ranges indicate that a cutoff value of < 9 mm for the Grabb–Oakes line [29, 39] may overestimate the risk of ventral compression of the brainstem and upper spinal cord. Of our 50 participants, 6–14% recorded measures ≥ 9 mm depending on the cervical position (Table 4).

Strengths of this study include that it is one of the largest studies to investigate reference CCI measures in different positions using udMRI. By including participants across a wide adult age range and of both sexes equally, we were able to identify differences in craniocervical joint mobility that occur naturally, permitting translation of our findings to the general population. Importantly, our study set the statistical significance level at *p* ≤ 0.005 accounting for family-wise error. Since a potential diagnosis of CCI can be a serious condition with significant financial and psychological ramifications, minimizing false-positive diagnoses was paramount.

Some limitations of this study should be acknowledged. The sample used for this study was retrospectively sourced from an imaging center. The participants were referred for udMRI for reasons other than CCI. While they did not have CCI symptoms, they did have symptoms related to pain or paresthesia for which their craniocervical region was imaged, constituting a possible selection bias. They were not symptom free and may display altered craniocervical kinematics compared to healthy individuals. Despite this, the BAA of 148.6° ± 10.3° in the neutral position agrees with a previous study reporting 148.9° ± 8.4° in a non-symptomatic sample [36]. Future studies should incept a cervical symptom-free cohort from which normative data and consequent cutoff criteria in neutral, and maximal flexion and extension using udMRI can be determined.

## Conclusions

This study provides reference values for four commonly used radiological measures used to assist in the diagnosis of craniocervical instability. As it is evident that people can demonstrate false-positive findings on udMRI using more conservative cutoff values, it is imperative to correlate these radiological measures with signs and symptoms of CCI. Special care needs to be taken when interpolating lines on MRI. Using standardized and detailed protocols with high-resolution imaging will further aid this task.
